# Coloboma of the Iris

**Published:** 1896-11

**Authors:** Richard H. Satterlee

**Affiliations:** 189 Delaware Avenue


### COLOBOMA OF THE IRIS.

By RICHARD H. SATTERLEE, M. D.

MR. L. S., employed on a railway, was sent to me for examination. His vision in the left eye was $\frac{20}{20}$; vision in the right eye was $\frac{20}{70}$. By tilting his head way back and still looking in the same direction as before, his vision was $\frac{20}{30}$ in the right eye.

Examination of the right eye showed a small pupil at the margin of the iris directly below the regular pupil. This extra pupil was arch-shaped, its base being parallel with the lower margin of the cornea. The pupil was nearly two millimeters in diameter and contracted under the influence of strong light the same as the larger pupil.

Vision with the lids wide open was only $\frac{20}{70}$, because the rays of light coming through the smaller pupil intercepted some of the rays coming through the normal pupil, resulting in a blurred image on the retina. Tilting the head far back covered the smaller pupil with the lower lid and allowed a normal condition of the eye to exist, and good vision resulted. A disc, with an aperture in the center about two millimeters in size, was placed directly in front of the eye. This cut off the rays of light from the smaller pupil. When this was placed before the eye it was not necessary to tilt the head back to get $\frac{20}{30}$ vision.

Attention is called to this test because in some cases of astigmatism the sight is much better if the head is tilted to one side or far back.

This man had never had the right eye injured, so we can eliminate traumatism. This condition is also mistaken for an iridotomy, but no history was given of such an operation, nor had there ever been any indication for an artificial pupil.

This case was unique in some respects, as the following com parison will show :

| *Coloboma of the iris generally is:* | *This case:* |
|---|---|
| Of hereditary transmission. | No such history. |
| Associated with cleft palate or fissnre of the eyelid. | No such lesions. |
| When unilateral left eye affected. | Right eye affected. |
| Marked astigmatism. | Almost no astigmatism. |
| Associated with congenital cataract. | Lens in healthy condition. |
| Amblyopic or nearly so. | Vision $\frac{20}{30}$. |
| Fundus hazy and indistinct. | Fundus healthy and media clear. |
| Involvement of retina and chor-oid. | Retina and choroid not in-volved. |
| Eye usually smaller than the other eye and imperfect in other respects. | Same size as the other eye and no other defect. |

The brief reference by most authorities to cases of this sort show that they are regarded merely as curiosities. The majority of eyes affected in this way have such poor sight that it is not worth while to operate. This case, on the contrary, would probably be improved by an operation. The test of tilting the head back and also the one of putting the perforated disc before the eye, both demonstrate that if the rays of light could be confined to the normal pupil the sight would be nearly perfect.

189 DELAWARE AVENUE.

